# Schistosomiasis and soil-transmitted helminths among an adult population in a war affected area, Southern Kordofan state, Sudan

**DOI:** 10.1186/1756-3305-5-133

**Published:** 2012-07-03

**Authors:** Alaa Hammad Ali Abou-Zeid, Tigani Abdullah Abkar, Rashid Osman Mohamed

**Affiliations:** 1 Public Health Department, Faculty of Medicine, Cairo University, Kasr Al Ainy St, Manial, Cairo, Egypt; 2 Ministry of Health, Kadugli, Sudan

**Keywords:** Schistosomiasis, Household, *S. haematobium*, *S. mansoni*, Soil-transmitted helminths, Southern Kordofan, Sudan

## Abstract

**Background:**

Schistosomiasis remains a major health problem at global and national levels, contributing to the vulnerability of the poor people in Sudan. Southern Kordofan is affected by Schistosomiasis but the disease prevalence was unknown.

**Methods:**

1826 adults were recruited in a community-based survey. Each recruited subject submitted at least 10 ml urine and one stool sample; they were also interviewed and filled in a questionnaire.

**Results:**

1826 adults were recruited in a community-based survey. Each recruited subject submitted at least 10 ml urine and one stool sample; they were also interviewed and filled in a questionnaire. The prevalence of *S. haematobium* was 6.9 % among the adult population. We estimated *S. mansoni* prevalence as 0.0 %. *S. haematobium* infection was focally distributed at the village level. The infection was associated with non preference of latrine use – if available, use of open water source for household affairs such as cleaning and also with the history of schistosomiasis treatment. The prevalence of soil transmitted helminths (STH) was also reported as high at 7.8 %, and two species were identified; *Hymenolepis nana* and *Giardia lamblia.*

**Conclusion:**

Schistosomiasis is a significant health problem among the adult population in Southern Kordofan. The estimated prevalence will serve as a guide in developing a Schistosomiasis Control Program and applying treatment plans.

## Background

Schistosomiasis remains one of the most prevalent parasitic diseases in the world with more than 200 million individuals infected, of whom over half suffer from related morbidity [1]. Whilst the global burden of schistosomiasis has been estimated at 1.7 to 4.5 million disability-adjusted life years [2,3], new research suggests this is a considerable underestimation of the ‘true’ burden of schistosomiasis [4,5]. However, schistosomiasis is a so-called neglected tropical disease, because it primarily affects poor rural communities in developing countries [6,7].

Sudan is one of the largest countries in Africa in terms of land surface area. The ongoing and protracted civil war, recurrent floods, droughts, storms, and the wide range of endemic, epidemic and epizoonotic diseases constitute important health and environmental threats [8]. Water ponds are the main characteristics of Southern Kordofan states where they stay wet for most of the year while open water sources constitute the main water source for households especially in rural areas. Generally speaking, the availability of latrines in the State is very low (less than 20 %) and the use of available latrines needs to be improved [9].

Early reports found that schistosomiasis is endemic in Sudan. However, few surveys have been conducted over the past 25 years because of the civil war [10]. Schistosomiasis has been identified as a real problem in the Southern Kordofan State. According to the National Schistosomiasis Control Program in Sudan [11], there are different treatment strategies including community mass treatment, selective treatment and case treatment. Due to the favorable disease ecology, we conducted this study to estimate the prevalence of schistosomiasis infection (both types) and identify associated risk factors among the adult population in Southern Kordofan to guide treatment strategies in the State.

## Methods

### Study area

**Figure 1:**
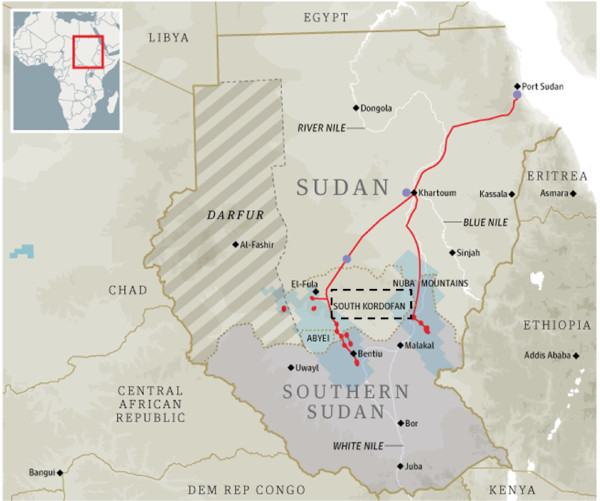
The study area showing the location of the South Kordofan (dotted rectangular) among other Sudanese states and other endemic bordering countries.

Southern Kordofan state (11°8′N 29°53′E) lies to the south of Sudan country bordering South Sudan (Figure 1). It is bordered by five states, North Kordofan from the north, White Nile from the north east, Upper Nile from the south east, Unity state from the south, South Darfur from the west and Bahr El-Gazal from the south east. The total surface area of the state is 970,470 km2 and the population is about 1,066,171 people. South Kordofan is divided into 9 localities and 29 administrative units. Rainfall is around 300–450 ml during the long rainy season (5 months) and the climate is Savanna.

The main activities of the communities in the state are farming and grazing. Water is provided mainly from dams, wells, Hafir (water collection behind a small wall erected by local community to collect rain water) and through water pumps. For more than 20 years, this state was the scene for war between north and southern Sudan that drastically affected the social and economic status of the State until now.

### Study design

This was a cross-sectional study in which urine and stool samples were collected from the adult population and examined to estimate the prevalence of schistosomiasis in SK State. The study was conducted in South Kordofan State (SK) where all 9 localities in the state were included. These localities are: Kadugli, Dilling (Middle part), Lagawa, Elsalam, Abyei, Kailak (Weastern part), Abu Gebeiha, Rashad and Talodi localities (Eastern part). Nuba Mountain Areas (under SPLA control) agreed to be included as part of the previously mentioned localities. In each locality there were 3–7 Administrative Units. The total number of Administrative Units in SK State is 40. Within each Administrative Unit, there are a variable number of villages and towns.

In this survey, the family was the sampling unit. A family was defined as: “a group of family members including siblings of only one wife (oldest) - if there was more than one wife- who was residing in the same house and sharing food from the same kitchen during the last three months”. We used a multi-stage random sampling technique to select families for the survey. From each locality, two Administrative Units were randomly selected and from each Administrative Unit, 2 villages/towns were randomly selected for the study, which made the total number of villages/towns included as 36 villages/towns. Within each village/town, we used a systematic random sampling technique to select 10 families within houses for the survey. Accordingly, once selected, the whole family members above the age of 18 years were invited to participate in the study. After obtaining the consent, the head of the family was interviewed to complete the family questionnaire while all family members were asked to give at least 10 ml urine and a stool sample each.

Inclusion criterion for villages/towns was that the village/town should have at least 50 families living inside (around 300 population). Inclusion criterion for families was any family that resided in the selected house for the past 6 months, while we excluded any severely ill family member at the time of the survey

### Sample size estimation

Sample size was estimated by using the WHO manual [12] for estimation of sample size for hypothesis testing for population proportion on the assumption that; a 5 % sampling error would be acceptable and 20 % schistosomiasis prevalence with a power at 90 %. This gave a sample size of 718 that was increased to 1826 to cater for design effect, nonresponse and missing data.

We conducted one-day training for the data collectors to ensure consistency of data collection. After submitting the consent, the family head was interviewed to fill in a structured pre-tested family questionnaire. From each recruited subject, at least 10 ml of urine and a stool sample were collected. Samples were stored on ice until transferred to the laboratory and then processed within the same day received. Two smears were prepared from each stool sample to test the presence of *S. mansoni* eggs using standard Kato Katz technique (WHO, 1994). The slides were read within 30 minutes of preparing them by a trained laboratory technician who was devoted to reading the slides. Urine was examined using the centrifugation method according to the Sudanese national standard.

### Statistical analysis

It is stated clearly that data was entered on Access database. Then, the database was converted to Statistical package for social sciences (SPSS) V. 13.0 for statistical analysis. Based on the study multistage design of the survey; weighting, stratification, and clustering were taken into account in all statistical analyses using complex sample analysis in SPSS to estimate sampling errors of estimators based on sample designs. The procedures estimate the variance from the variation among the villages/towns and pool stratum variance estimates to compute the overall variance estimate. A weighting factor was used in the analysis to reflect the likelihood of sampling each family in a stratum. The weight used for estimation is given by the following formula: W = W1 x W2 x W3. Where W1 = the inverse of the probability of selecting the administrative unit in a strata, W2 = the inverse of the probability of selecting the village/town in a stratum and, W3 = the inverse of the probability of selecting a family from the villages/towns.

Quantitative variables such as age and density of *S. haematobium* infection were converted into categorical variables using suitable cut off points. The prevalence and 95 % confidence interval (95 % CI) of the *S. haematobium* infection were computed. Bivariate analysis for the relationship between the prevalence of *S. haematobium* infection and various risk factors that may be associated with infection was conducted using Pearson Chi square test. Pearson correlation coefficient was estimated for the correlation between ova density in urine and age. The recorded P value for the Chi Square test was used to build models for the multivariate analysis where only variables with p < 0.02 were included. Logistic regression model with backward elimination for variables that had association with *S. haematobium* at a significant level of p value <0.05 were retained in the model. Crude odds ratio (OR) with the 95 % CI were calculated and reported for the association between the prevalence of *S. haematobium* infection and risk factors.

### Ethical clearance

This study was conducted using protocols and tools approved by the Sudanese Federal Ministry of Health. Ethical approval was obtained from the SK State Ministry of Health. Approvals of traditional community leaders were obtained before conducting the survey in any village/town. The whole adult family members were informed before starting the procedures of the survey and if agreed, they were recruited by signing/stamping the consent form.

## Results

In this survey, 1826 adults were recruited from all 9 localities in Southern Kordofan (SK) State. These subjects were recruited from 36 villages/towns in 18 administrative Units in the state.

All subjects submitted urine samples while only 72.0 % of adults submitted stool samples. Urine samples were examined for the presence of *Schistosoma haematobium* while stool samples were examined for the presence of *Schistosoma mansoni* infection.

Most of the adults recruited in the household survey were from rural areas (78.3 %). The mean age of recruited adults in the community survey was 33.5 ± 15.7 years. Subjects were mainly females (62.7 %) represented in the family as daughters (25.9 %) and mothers (17.1 %). Subjects were mostly illiterate (54.4 %) and the unemployed including housewives represented 48.4 % while the rest were mainly farmers (30.5 %) (Table 1).

**Table 1:** Some demographic characteristics of the adults recruited in the household survey, SK survey, Sudan, 2009

		**No (n = 1826)**	**%**
Location	*Urban*	379	21.7
	*Rural*	1429	78.3
Age	*≤ 20 yrs*	498	27.3
	*21-40 yrs*	833	45.6
	*>40 yrs*	495	27.1
Gender	*Male*	682	37.3
	*Female*	1144	62.7
Relation in the Family	*Father*	235	12.9
	*Mother*	312	17.1
	*Suns*	306	16.8
	*Daughter*	473	25.9
	*Relative*	500	27.4
Education	*Illiterate*	994	54.4
	*Primary*	591	32.4
	*Intermediate*	87	4.8
	*Secondary*	118	6.5
	*University*	36	2.0
Occupation	*Student*	152	8.3
	*Unemployed/Housewife*	884	48.4
	*Farmer*	557	30.5
	*Trader*	43	2.4
	*Gov employee*	60	3.3
	*Military*	70	3.8
	*Pasturer*	17	0.9
	*Manual worker*	43	2.4

*S. haematobium* eggs were found in 6.9 % (95 % CI 5.7 – 8.1 %). The overall ova density among the infected population was 15.7 ± 12.6 (eggs/10 ml urine). Only 2/126 subjects had severe *S. haematobium* infection indicated by ova density ≥ 50 eggs/10 ml urine (Table 2). The prevalence of *S. mansoni* was 0.0 % among the studied population. The prevalence of soil transmitted helminths (STH) was also reported as high at 7.8 %, and two species were identified; *Hymenolepis nana* and *Giardia lamblia.*

**Table 2:** Schistosomiasis infection and soil-transmitted helminths among adults recruited in the household survey, SK survey, Sudan, 2009

**Variable**		**Adults**	
		**No (n = 1826)**	**%**
*S. hematobium* infection	*Positive*	126	6.9
		*Mean*	*±SD*
Severity of *S. hematobium* infection	*(eggs/10 ml urine)*	15.7	*±*12.6
*S. mansoni* infection*	*Positive*	0/1315	0.0 %
Soil-transmitted helminths*	*Positive*	102/1315	7.8 %

*Not all subjects submitted stool samples.

Prevalence of *S. haematobium* among adults differed by geographic location at the locality, administrative unit and village/town levels where the variation became more noticeable moving from the locality to the village/town direction. Prevalence ranged from 0.0 % - 19.2 % among the 9 localities but the range was from 0.0 % - 24.0 % among the 18 administrative units and was from 0.0 % - 43.1 % among villages/towns. The prevalence in 18 out of 36 studied villages/towns was 0.0 %. Only 7 villages/towns had prevalence above 10 % while only 4 had prevalence above 20 %.

Infection was higher in rural areas (7.6 %) compared to urban areas (4.3 %). In addition, infection was much higher in the Western part of the State (12.5 %) compared to 3.3 % in the Eastern part and 0.0 % in the Middle part of the State (Table 3).

**Table 3:** General demographic characteristics and factors associated with

**Variable**		**Total**		***S. haematobium *infected **	***χ***^**2**^	**Total**
		**No (n = 1826)**	**%**	**No (n = 126)**	**%**	
Location	*Urban*	379	21.7	13	4.3	0.019
	*Rural*	1429	78.3	109	7.6	
Geographic part within the State	*Middle*	415	22.7	0	0	<0.001
	*Eastern*	550	30.1	18	3.3	
	*Western*	861	47.2	108	12.5	
Relation in the Family	*Father*	235	12.9	9	3.8	0.016
	*Mother*	312	17.1	34	10.9	
	*Suns*	306	16.8	23	7.5	
	*Daughter*	473	25.9	30	6.3	
	*Relative*	500	27.4	30	6.0	
Gender	*Female*	1144	62.7	93	8.1	0.007
	*Male*	682	37.3	33	4.8	
Age	*≤ 20 yrs*	498	27.3	36	7.2	0.45
	*21-40 yrs*	833	45.6	51	6.1	
	*>40 yrs*	495	27.1	39	7.9	
Education	*Above primary*	241	13.2	6	2.5	0.014
	*Primary*	591	32.4	43	7.3	
	*Illiterate*	994	54.4	77	7.7	
Occupation	*Other*	385	21.1	12	3.1	<0.001
	*Unemployed/Housewife*	884	48.4	60	6.8	
	*Farmer*	557	30.5	54	9.7	
Source of water	*Closed (pipe/Pump)*	1005	55.0	59	5.9	0.06
	*Open*	821	45.0	67	8.2	
Latrines	*Available*	1013	55.5	65	6.4	0.36
	*Not available*	813	44.5	61	7.5	
Latrine for use	*Preferred*	927	50.8	52	5.6	0.033
	*Not preferred*	899	49.2	74	8.2	
Distance from home to nearest open water source	*≥ 1 Km*	215	11.8	0	0.0	<0.001
	*< 1 Km*	1611	88.2	126	7.8	
Household use (washing) of open water sources	*No*	490	26.8	9	1.8	<0.001
	*Yes*	1336	73.2	117	8.8	
Personal use (Bath/swim) of open water sources	*No*	406	22.2	9	2.2	<0.001
	*Yes*	1420	77.8	117	8.2	
Health facility in the same village	*Present*	1043	57.1	58	5.6	0.01
	*Absent*	783	42.9	68	8.7	
History of Schistosomiasis treatment in	*No*	1762	96.5	113	6.4	<0.001
	*Yes*	64	3.5	13	20.3	

*Schistosoma haematobium* infection among subjects recruited in the household survey, SK, Sudan, 2009.

Multivariate analysis showed that living in the Western part carries almost 4 times the risk of infection compared to the Eastern part of the State (OR 3.83; 95 % CI 2.28-6.42) (Table 4).

**Table 4:** **Bivariate and adjusted association of *Schistosoma haematobium *by risk factors among subjects recruited in the household survey, SK, Sudan 2009**

**Variable**		**Crude**	**Adjusted**
		**OR (95 % CI)**	**OR (95 % CI)**
Location	*Urban*	1	Removed
	*Rural*	1.85 (1.09-3.12)	
Geographic part within the State	*Middle*	-	-
	*Eastern*	1	1
	*Western*	4.24 (2.5-7.1)	3.83 (2.28-6.42)
Relation in the Family	*Father*	1	Removed
	*Mother*	3.07 (1.44-6.54)	
	*Suns*	2.04 (0.93-4.50)	
	*Daughter*	1.70 (0.79-3.64)	
	*Relative*	1.60 (0.75-3.43)	
Gender	*Female*	1	1
	*Male*	0.58 (0.38-0.87)	0.61 (0.40-1.94)
Age	*≤ 20 yrs*	1	Not included
	*21-40 yrs*	0.84 (0.54-1.30)	
	*>40 yrs*	1.10 (0.69-1.69)	
Education	*Above primary*	1	Removed
	*Primary*	3.07 (1.29-7.32)	
	*Illiterate*	3.29 (1.42-7.64)	
Occupation	*Other*	1	Removed
	*Unemployed/Housewife*	2.26 (1.20-4.26)	
	*Farmer*	3.34 (1.76-6.33)	
Source of water	*Closed (pipe/Pump)*	1	Removed
	*Open*	1.43 (0.99-2.05)	
Latrines	*Available*	1	Not included
	*Not available*	1.18 (0.82-1.70)	
Latrine use	*Preferred*	1	1
	*Not preferred*	1.51 (1.05-2.18)	1.63 (1.11-2.41)
Distance from home to nearest open water source	*≥ 1 Km*	1	Removed
	*< 1 Km*	1.09 (1.07-1.10)	
Household utilization of open water sources	*No*	1	1
	*Yes*	5.13 (2.58-10.19)	2.10 (1.03-4.28)
Personal utilization (Bath/swim) of open water sources	*No*	1	Removed
	*Yes*	3.96 (1.99-7.88)	
Health facility in the same village	*Present*	1	Removed
	*Absent*	1.62 (1.12-2.32)	
History of Schistosomiasis treatment	*No*	1	1
	*Yes*	3.72 (1.97-7.04)	4.19 (2.10-8.37)

*S. haematobium* infection was found to be the highest among mothers (10.9 %) and lowest among fathers (3.8 %) (p = 0.016). Similarly, prevalence was found to be significantly higher (p = 0.007) among females (8.1 %), compared to males (4.8 %). Prevalence of infection was similar among those aged ≤ 20 years compared to other age groups (p = 0.45). Illiteracy was associated with higher prevalence of infection (7.7 %) when compared to having received education above the primary level (2.5 %) (p = 0.014). Being a farmer was associated with higher prevalence of infection (9.7 %) compared to those who do not contact water as part of their routine work (3.1 %) (p < 0.001).

We could not estimate differences in infection associated with availability of latrines (p = 0.36). However, infection was significantly less among those who preferred using latrines – if available – (5.6 %) compared to those who preferred not (8.2 %) (p = 0.033).

The usage of open water sources (8.2 %) was weakly associated with infection (p = 0.06) compared to using pipe water or hand pumps (closed). Living within 1 km close to an open water source (7.8 %) was associated with higher infection (p < 0.001) compared to those who lived > 1 km away from an open water source (0.0 %). Using water from an open source for household affairs such as cleaning and for bathing/swimming was also associated with higher prevalence of infection (p < 0.001) (Table 3).

Presence of a health facility in the same location was associated with lower prevalence of infection (p < 0.01). Prevalence was 20.3 % among those who had a history of schistosomiasis treatment compared to only 6.4 % among those who had no previous history of treatment (p < 0.001) (Table 3).

Multivariate analysis showed significant association between *S. haematobium* infection and the following risk factors: not preferring latrine use – if available – was associated with more than 1.5 times greater risk for (OR 1.63; 95 % CI 1.11-2.41), use of open water source for household affairs such as cleaning carried 2 times the risk of infection (OR 2.10; 95 % CI 1.03-4.28) and lastly history of schistosomiasis treatment was associated with more than 4 times the risk compared to not having received treatment (OR 4.19; 95 % CI 2.10-8.37) (Table 4).

Only 15/126 (11.9 %) reported passing red urine, so self reporting of passing red urine was insensitive as a predictor of *S. haematobium* infection among the studied population. Self reporting of passing red urine showed much better specificity (95.9 %) compared to sensitivity. Sensitivity was better in males (33.3 %) compared to females (4.3 %).

The predictive value positive for reporting passing red urine was as low as 17.9 % among subjects. On the other hand, the negative predictive value was much better at 93.6 %. The overall accuracy of self reporting of passing red urine was 90.0 % among the studied population.

## Discussion

The aim of this study is to understand the prevalence of Schistosomiasis, and soil-transmitted helminths in a war affected area of Sudan. The infection of schistosomiasis, and soil-transmitted helminths (STH) has been a matter of great public health concern throughout Africa, and the world for decades [13-15]. To our knowledge, this is the first State wide household survey for Schistosomiasis in Southern Kordofan State. The prevalence of *S. haematobium* infection was 6.9 % among adults recruited in this community survey. We found that S. mansoni infection does not exist among the adult population in SK State (prevalence was 0.0 %). The prevalence of *S. haematobium* varied greatly by locality. The Western part of the state had a significantly higher rate of infection compared to the Middle and Eastern parts. Among adults, *S. haematobium* infection was associated with refusing latrine use if available, use of open water sources for household purposes such as washing and lastly with a history of schistosomiasis treatment.

Previous old data reported that the prevalence of *S. haematobium* ranged from 0.4 - 44 % and S. mansoni 4.8 - 6.8 % in different parts of Southern Sudan [16]. In more recent studies, authors reported prevalence of *S. haematobium* and *S. mansoni* in White Nile State as 21.4 % and 10.1 % respectively [17]. In Southern Sudan, *S. haematobium* and *S. mansoni* was prevalent among 2.5 % & 1.9 % of population respectively [11].

The difference in prevalence rates among localities is similar to the findings in White Nile and many other studies inside and outside Sudan [17,18]. Our estimation for the severity of infection in SK (0.2 %) is within the range mentioned by other studies [11].

The association between gender and *S. haematobium* infection varied in different communities. In our study, we could not estimate an association between infection and gender. Other studies in White Nile and in Southern Sudan also could not estimate such an association [11,17].

A survey conducted at a large number of sites throughout Sudan in 1994 examined 2489 fecal samples and found 53 samples positive for STH. The conclusion of the FMoH is that the cumulative prevalence of infection with at least one STH ranged from 10-35 % with the majority of infection in Southern Sudan. In our study, the prevalence of STH was 7.8 %, which is close to the above-mentioned findings [19].

The prevalence of *S. haematobium* infection was not associated with age in SK, which was similar to the findings in the White Nile survey [17]. Similarly, we could not estimate association between education and occupation on the one hand and prevalence of *S. haematobium* infection on the other hand. This finding is similar to the finding reported in Malawi [18]. However; conflicting results have been reported from other studies for such relationships [20,21]. These findings could be attributed to the fact that people in SK depend heavily on open water sources for different purposes with all populations similarly exposed.

Proximity of open water sources has been consistently reported as associated with *S. haematobium* infection in many studies [22,23]. Interestingly, we could not estimate an association between home proximity to open water sources and *S. haematobium* infection, which is consistent with other studies [18]. Our finding can be explained by the fact that open water sources remain necessary for people to use regardless of the distance.

History of urinary schistosomiasis treatment was strongly associated with *S. haematobium* infection in SK, which is similar to other findings in Malawi [18]. This could be explained by the fact that communities with high prevalence rates tend to cluster around contaminated water sources with continuous exposure even after treatment [24].

The specificity of self reporting of passing red urine in our study was consistent with the specificity reported from other studies ranging from 58-96 % [25,26]. However, the sensitivity we reported (11.9 %) was less than the average reported in the same previously mentioned studies (50-100 %). Possible reasons could be that students were afraid to be referred to hospital for expensive treatment if they reported passing red urine. These results could have been verified if we conducted Circulating Anodic Antigen (CAA) to compare it to self-reporting of passing red urine.

## Conclusion

*S. haematobium* is a significant health problem affecting the health of the vulnerable population in Southern Kordofan State. Our study proved that S. mansoni does not exist among the different population groups. The use of a questionnaire as a screening tool for *S. haematobium* infection is not valuable due to its low sensitivity compared to urine examination for *S. haematobium* eggs, which will remain the gold standard test for diagnosing the infection.

Based on the findings of this survey, the Schistosomiasis Control Program in SK State within the SMoH shall direct resources to combat schistosomiasis with all focus on *S. haematobium*. This study illustrates the national efforts to better control schistosomiasis activities by fostering the Schistosomiasis Control Programs at the State level in Sudan. The SMoH shall consider targeted/selective treatment in villages/towns in SK based on prevalence estimates (National Schistosomiasis Control Program, 2006). Repetition of this survey in SK State is recommended to update the treatment policy of the SMoH. Similar State-wide surveys targeting younger and school children are also recommended to guide treatment for other population groups as well. In addition, studies on different treatment schemes that may guide better policies to ensure timely and better utilization of praziquantel is recommended.

## Competing interests

The author declares that they have no competing interests.

## Authors’ contributions

AA-Z carried out the work and analyzed the data and wrote the manuscript. The other two authors helped in the field and experimental work and helped in drafting the manuscript. All authors read and approved the final version of the manuscript.

## References

[B1] Steinmann P Keiser J Bos R Tanner M Utzinger J Schistosomiasis and water resources development: systematic review, meta-analysis, and estimates of people at risk Lancet Infect Dis 2006 6 411 425 10.1016/S1473-3099(06)70521-7 16790382

[B2] WHO: Prevention and control of schistosomiasis and soil-transmitted helminthiasis: report of a WHO expert committee WHO Tech Rep Ser No 2002 912 1 57 12592987

[B3] WHO The world health report: changing history 2004 World Health Organization, Geneva

[B4] King CH Dickman K Tisch DJ Reassessment of the cost of chronic helmintic infection: a meta-analysis of disability-related outcomes in endemic schistosomiasis Lancet 2005 365 1561 1569 10.1016/S0140-6736(05)66457-4 15866310

[B5] Jia TW Zhou XN Wang XH Utzinger J Steinmann P Wu XH Assessment of the age-specific disability weight of chronic schistosomiasis japonica Bull World Health Organ 2007 85 458 465 10.2471/BLT.06.033035 17639243 PMC2636356

[B6] Hotez PJ Molyneux DH Fenwick A Kumaresan J Ehrlich SS Sachs JD Savioli L Control of neglected tropical diseases N Engl J Med 2007 57 1018 1027 17804846 10.1056/NEJMra064142

[B7] Keiser J Utzinger J Advances in the discovery and development of novel trematocidal drugs Expert Opin Drug Discov 2007 2 9 23 10.1517/17460441.2.S1.S9 23489037

[B8] WHO Country Cooperation Strategy for WHO and Sudan 2008–2013 2009 a Joint Strategy by WHO and Sudan Federal Ministry of Health, WHO EM/ARD/032/E

[B9] Federal Ministry of Health The Sudan Household Health Survey 2007 Report from the Federal Ministry of Health, Sudan

[B10] Roberto D Mario C Claudio B Otine D Oyugi V Montresor A Schistosoma haematobium and S. mansoni among Children, Southern Sudan. Emerging Infectious Diseases 2007 13 1504 1506 10.3201/eid1310.070356 PMC2851532 18257996

[B11] Kurti I Othman H Guidelines for distribution of treatment for Bilharzias and soil transmitted parasites 2006 Federal Ministry of Health guide, National Schistosomiasis Control Program 6 8

[B12] Lwanga S Lemeshow S Sample size Determination in health studies: a practical manual 1991 World Health, Organization 2 31

[B13] Opisa S Odiere MR Jura WG Karanja DM Mwinzi PN Malacological survey and geographical distribution of vector snails for schistosomiasis within informal settlements of Kisumu City, western Kenya Parasit Vectors 2011 7 4 226 10.1186/1756-3305-4-226 PMC3247081 22152486

[B14] Ahmed A Al-Mekhlafi HM Choy SH Ithoi I Al-Adhroey AH Abdulsalam AM Surin J The burden of moderate-to-heavy soil-transmitted helminth infections among rural malaysian aborigines: an urgent need for an integrated control programme Parasit Vectors 2011 30 4 242 10.1186/1756-3305-4-242 PMC3259102 22208559

[B15] Sun LP Liang YS Wu HH Tian ZX Dai JR Yang K Hong QB Zhou XN Yang GJ A Google Earth-based surveillance system for schistosomiasis japonica implemented in the lower reaches of the Yangtze River, China Parasit Vectors 2011 27 4 223 10.1186/1756-3305-4-223 PMC3250965 22117601

[B16] Amir MA Omer AHS Schistosoma haematobium infection at El Ghorashi Diary farm, Khartoum, Sudan Sudan Med J 1972 10 194 201

[B17] Ahmed ES Daffalla A Christensen NO Madsen H Patterns of infection and transmission of human schistosomiasis mansoni and schistosomiasis haematobium in White Nile Province, Sudan Ann Trop Med Parasitol 1996 90 173 80 8762407 10.1080/00034983.1996.11813041

[B18] Kapito-Tembo AP Mwapasa V Meshnick SR Samanyika Y Banda D Bowie C Radke S Prevalence Distribution and Risk Factors for Schistosoma haematobium Infection among School Children in Blantyre, Malawi PLOS NTD 2009 3 1 8 10.1371/journal.pntd.0000361 PMC2614474 19156193

[B19] Richer M Ruiz JA Brooker S Kolaczinski JH Neglected Tropical Diseases And Their Control In Southern Sudan Situation Analysis, Intervention Options Appraisal And Gap Analysis. Ministry of Health, Government of Southern Sudan: Soil Transmitted Helminths (STHs) 2008 http://www.malariaconsortium.org/userfiles/file/NTD%20Resources/ntds_southern_sudan%5B1%5D.pdf

[B20] Gazzinelli A Velasquez-Melendez G Crawford SB LoVerde PT Correa-Oliveira R Socioeconomic determinants of schistosomiasis in a poor rural area in Brazil Acta Trop 2006 99 260 271 10.1016/j.actatropica.2006.09.001 17045559 PMC1828742

[B21] de Cassia Ribeiro Silva R Barreto ML Assis AM de Santana ML Parraga IM The relative influence of polyparasitism, environment, and host factors on schistosome infection Am J Trop Med Hyg 2007 77 672 675 17978069

[B22] Clennon JA King CH Muchiri EM Kariuki HC Ouma JH Spatial patterns of urinary schistosomiasis infection in a highly endemic area of coastal kenya Am J Trop Med Hyg 2004 70 443 448 15100462

[B23] Handzel T Karanja DM Addiss DG Hightower AW Rosen DH Geographic distribution of schistosomiasis and soil-transmitted helminths in western kenya: Implications for anthelminthic mass treatment Am J Trop Med Hyg 2003 69 318 323 14628951

[B24] Clennon JA Mungai PL Muchiri EM King CH Kitron U Spatial and temporal variations in local transmission of schistosoma haematobium in msambweni, kenya Am J Trop Med Hyg 2006 75 1034 1041 17172362

[B25] Lengeler C Utzinger J Tanner M Questionnaires for rapid screening of schistosomiasis in sub-saharan africa Bull World Health Organ 2002 80 235 242 11984610 PMC2567742

[B26] von ST MMA Utzinger J N’Goran EK Control of urinary schistosomiasis: An investigation into the effective use of questionnaires to identify high-risk communities and individuals in Niger state, Nigeria Trop Med Int Health 2000 5 53 63 10.1046/j.1365-3156.2000.00508.x 10672206

